# The Obesity–OSA–Arrhythmia Axis: Pathophysiological Mechanisms and Translational Therapeutic Targets

**DOI:** 10.3390/life16050737

**Published:** 2026-04-29

**Authors:** Fulvio Cacciapuoti, Ilaria Caso, Antonietta Buonomo, Salvatore Crispo, Vittorio Taglialatela, Gerardo Carpinella, Mario Volpicelli, Ciro Mauro

**Affiliations:** 1 Division of Cardiology, “Antonio Cardarelli” Hospital, 80131 Naples, Italy; 2 Department of Cardiology, “Vincenzo Monaldi” Hospital, 80131 Naples, Italy; 3 Department of Cardiology, “Pellegrini” Hospital, 80134 Naples, Italy; 4 Division of Electrophysiology, “Santa Maria della Pietà” Hospital, 80026 Naples, Italy

**Keywords:** obstructive sleep apnea, obesity, atrial fibrillation, cardiac arrhythmias, epicardial adipose tissue, tirzepatide, cardioneuroablation, autonomic dysfunction

## Abstract

Obesity and obstructive sleep apnea (OSA) frequently coexist and synergistically contribute to cardiovascular disease through interconnected mechanical, metabolic, and autonomic mechanisms. This interplay promotes myocardial electrical instability and structural remodeling, favoring the development and persistence of cardiac arrhythmias, particularly atrial fibrillation. Among the key mediators linking obesity to arrhythmogenesis, epicardial adipose tissue has emerged as a relevant factor that may contribute to local pro-inflammatory, pro-fibrotic, and autonomic effects on the myocardium. In parallel, OSA-related intermittent hypoxia and intrathoracic pressure swings further amplify electrical instability and autonomic imbalance, reinforcing a self-sustaining arrhythmogenic substrate. Therapeutic strategies are increasingly shifting toward upstream interventions targeting these underlying mechanisms. Metabolic therapies, including the dual GIP/GLP-1 receptor agonist tirzepatide, have demonstrated substantial weight reduction and improvement in OSA severity, with potential indirect benefits on arrhythmic risk through modulation of visceral adiposity, inflammation, and metabolic dysfunction. On the electrophysiological side, cardioneuroablation has emerged as a potentially investigational option in selected patients with vagally mediated bradyarrhythmias, although its role remains to be fully defined. Overall, these observations support an integrated, phenotype-driven approach combining respiratory therapy, metabolic modulation, and targeted electrophysiological interventions. This framework may help redefine therapeutic priorities, shifting from symptom control toward modification of the underlying arrhythmogenic substrate and improvement of long-term cardiovascular outcomes.

## 1. Introduction

Obesity, obstructive sleep apnea (OSA), and cardiac arrhythmias frequently coexist and interact in ways that amplify both cardiometabolic and electrophysiological risk. As obesity becomes increasingly prevalent worldwide, sleep-disordered breathing—particularly OSA—has emerged as a common comorbidity in patients with cardiovascular disease. In this setting, atrial fibrillation represents the most frequent arrhythmic manifestation, with prevalence rates approaching 40–50% in selected populations [1]. In everyday clinical practice, the coexistence of these conditions is often under-recognized, leading to fragmented management strategies. Current guidelines emphasize the importance of addressing modifiable risk factors, including obesity and sleep-disordered breathing, in the management of atrial fibrillation [2].

The pathophysiological links between OSA and arrhythmias are multifactorial and involve the interplay of mechanical stress, metabolic dysfunction, and autonomic imbalance. Recurrent upper-airway obstruction in OSA promotes intermittent hypoxia and mechanical stress, leading to oxidative imbalance, inflammation, and early electrical remodeling [3].

In parallel, negative intrathoracic pressures increase cardiac afterload and atrial stretch, further destabilizing electrical conduction. These effects are particularly relevant in patients with heart failure with preserved ejection fraction (HFpEF), in whom OSA is highly prevalent and contributes to impaired ventricular relaxation and pulmonary pressure fluctuations [4].

Beyond these mechanisms, obesity itself plays a central role in shaping the arrhythmogenic substrate. Among obesity-related factors, epicardial adipose tissue (EAT) has emerged as a relevant mediator that may link metabolic dysfunction to myocardial electrical remodeling [5].

**Figure 1:**
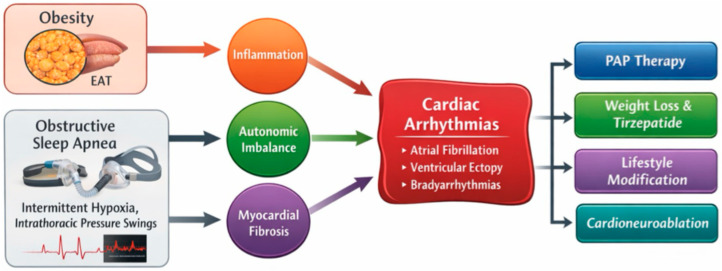
Schematic representation of the interplay between obesity, obstructive sleep apnea (OSA), and cardiac arrhythmias. Obesity-related epicardial adiposity and OSA-induced intermittent hypoxia promote inflammation, autonomic imbalance, and myocardial fibrosis, leading to increased arrhythmic risk. Therapeutic strategies include PAP therapy, weight reduction, metabolic treatment, and cardioneuroablation.

Collectively, these processes define a self-reinforcing “obesity–OSA–arrhythmia axis,” in which metabolic, respiratory, and electrophysiological factors converge and may contribute to disease progression (Figure 1).

In recent years, growing attention has been directed toward therapeutic strategies capable of interrupting this cycle. Beyond positive airway pressure therapy and lifestyle interventions, metabolic treatments—including incretin-based therapies—have shown the potential to reduce body weight, visceral adiposity, and OSA severity. In parallel, advances in electrophysiological techniques and autonomic modulation have expanded the range of options available for selected patients.

The heterogeneity of patients within the obesity–OSA–arrhythmia axis deserves particular attention. Rather than representing a uniform pathophysiological entity, this condition may encompass distinct clinical phenotypes characterized by the predominance of metabolic, respiratory, or autonomic mechanisms. Recognizing these phenotypic differences may have important implications for both risk stratification and therapeutic decision-making. A phenotype-driven framework may therefore provide a more clinically actionable model, allowing the integration of targeted interventions aimed at the dominant pathophysiological drivers in individual patients.

This review provides an integrated overview of the mechanisms linking obesity, OSA, and arrhythmias, with a focus on emerging metabolic and electrophysiological therapeutic strategies.

In addition, the obesity–OSA–arrhythmia axis may be interpreted not only as the coexistence of interacting clinical conditions but also as a systems-level network sustained by persistent metabolic stress, intermittent hypoxia, autonomic imbalance, and chronic low-grade inflammation. Within this framework, oxidative stress and inflammation should not be viewed exclusively as downstream markers of tissue injury but also as dynamic regulatory processes that may help organize and perpetuate disease chronicity. This broader perspective may support a more integrated interpretation of myocardial remodeling and arrhythmogenesis across different clinical phenotypes.

## 2. Visceral Obesity, Epicardial Adipose Tissue, and Arrhythmogenesis

EAT is increasingly recognized as a relevant component linking obesity to arrhythmogenesis through direct anatomical and functional interactions with the myocardium. Unlike other fat depots, EAT is anatomically contiguous with the myocardium and coronary arteries, without a fascial barrier, allowing for direct paracrine and vasocrine interactions with the underlying cardiac tissue.

In the setting of obesity, EAT undergoes both quantitative expansion and qualitative transformation, shifting toward a pro-inflammatory and pro-fibrotic phenotype. This is characterized by increased secretion of cytokines such as interleukin-6 and tumor necrosis factor-α, along with adipokines and free fatty acids, which promote oxidative stress, endothelial dysfunction, and myocardial fibrosis [6]. These alterations may contribute to the development of an arrhythmogenic substrate, as suggested by histological and imaging studies reporting associations between increased EAT volume, atrial fibrosis, and conduction heterogeneity [7]. In particular, peri-atrial and peri-pulmonary vein fat depots appear to play a relevant role in modulating local electrophysiological properties and facilitating reentry mechanisms.

**Figure 2:**
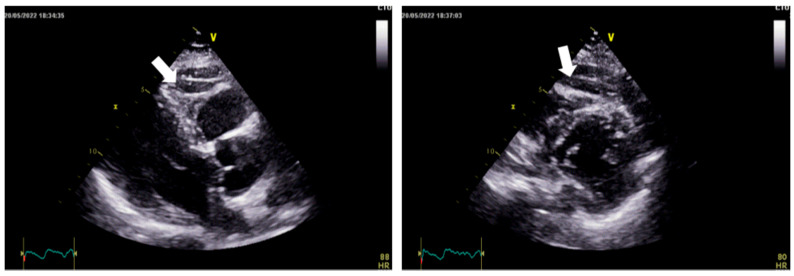
Echocardiographic assessment of epicardial adipose tissue. Parasternal long-axis views showing epicardial adipose tissue (EAT) as an echo-lucent space between the right ventricular free wall and the visceral pericardium (arrows).

The assessment of EAT has therefore gained increasing clinical relevance, particularly in the context of visceral obesity and arrhythmic risk stratification. Transthoracic echocardiography provides a rapid and widely accessible method to estimate EAT thickness. It is typically measured in the parasternal long-axis view as the echo-lucent space between the right ventricular free wall and the pericardium (Figure 2) [8]. However, its ability to fully characterize epicardial fat distribution is limited, as posterior fat depots cannot be reliably visualized and measurements may be influenced by acoustic window quality. In addition, advanced echocardiographic parameters, such as left atrial strain and tissue Doppler-derived conduction time (PA-TDI), provide indirect but clinically meaningful markers of atrial remodeling and fibrosis, reflecting the electrophysiological impact of EAT on the atrial substrate.

In contrast, cardiac computed tomography allows for more accurate and reproducible quantification of EAT volume, offering a comprehensive three-dimensional evaluation and demonstrating significant associations with coronary artery disease burden and overall cardiovascular risk [9]. The integration of imaging modalities therefore enables a more complete phenotypic characterization, supporting both risk stratification and individualized therapeutic decision-making.

Beyond structural remodeling, EAT may also influence cardiac autonomic function. Its close relationship with the intrinsic cardiac nervous system, including ganglionated plexi, suggests a possible contribution to autonomic dysregulation, a relevant feature of arrhythmogenesis in OSA. Increased EAT volume has been associated with enhanced sympathetic activity and altered vagal modulation, further contributing to electrical instability [10]. In patients with OSA, these mechanisms are further amplified, as intermittent hypoxia and sleep fragmentation promote EAT inflammation and metabolic dysfunction, reinforcing the link between respiratory disturbance and myocardial remodeling.

Importantly, EAT represents a modifiable component of cardiometabolic risk. Lifestyle interventions, including caloric restriction, weight reduction, and regular physical activity, have been associated with reductions in EAT volume and inflammatory activity. Exercise, in particular, improves insulin sensitivity and promotes anti-inflammatory pathways, contributing to a more favorable metabolic and electrophysiological profile [11]. In patients with atrial fibrillation, structured lifestyle modification has been shown to reduce arrhythmic burden and improve outcomes, particularly when integrated into a comprehensive and multidisciplinary management strategy [12].

Taken together, EAT should be regarded not merely as a marker of cardiometabolic risk but as a biologically active tissue that may contribute to arrhythmogenesis. However, much of the evidence linking EAT to arrhythmogenesis remains observational, and the extent to which EAT directly contributes to arrhythmic outcomes, rather than reflecting broader cardiometabolic risk, remains to be fully clarified.

## 3. Obstructive Sleep Apnea and Arrhythmias

OSA is a well-recognized trigger of both supraventricular and ventricular arrhythmias, with manifestations closely linked to the timing of respiratory events.

Atrial fibrillation (AF) represents the most frequent arrhythmia in this setting and is characterized by both increased incidence and higher recurrence rates after rhythm control strategies. In patients with untreated OSA, AF tends to be more persistent and less responsive to catheter ablation, suggesting that ongoing respiratory disturbance contributes to the maintenance of the arrhythmogenic substrate [13]. Beyond AF, OSA is associated with a broad spectrum of rhythm disturbances. Ventricular ectopy and non-sustained ventricular tachycardia are commonly observed during sleep, often clustering around apneic episodes and periods of oxygen desaturation. These arrhythmias are typically transient but may become clinically significant in patients with structural heart disease or increased arrhythmic susceptibility [14].

**Figure 3:**
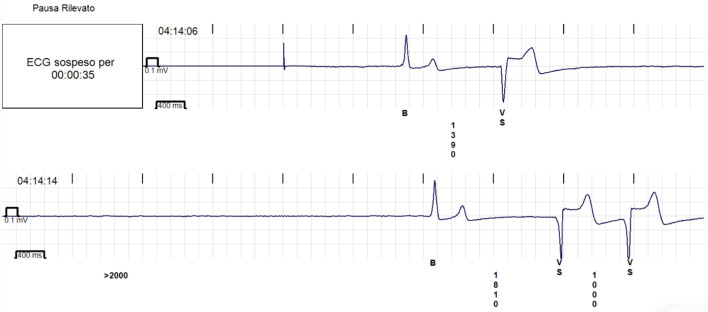
Nocturnal bradyarrhythmia detected by a loop recorder. Representative tracing showing a prolonged sinus pause during sleep. Such bradyarrhythmias are commonly observed in patients with obstructive sleep apnea and reflect transient vagally mediated conduction disturbances associated with apneic events.

Bradyarrhythmias represent another distinctive feature of OSA. Sinus pauses, sinus bradycardia, and varying degrees of atrioventricular block are frequently recorded during sleep and are usually temporally linked to apneic events (Figure 3). In most cases, these disturbances are functional and reflect exaggerated vagal activation rather than intrinsic conduction system disease. Their recognition is clinically important, as appropriate treatment of OSA often leads to resolution or a marked reduction in these abnormalities [15].

Not all arrhythmias observed in this setting require invasive treatment, and careful clinical evaluation is essential to avoid unnecessary interventions. From a clinical perspective, the key challenge lies in distinguishing reversible, apnea-related rhythm disturbances from arrhythmias sustained by fixed structural or electrical substrates (Table 1).

Continuous rhythm monitoring, combined with polysomnographic data, can help establish a temporal correlation between respiratory events and arrhythmias, guiding therapeutic decisions.

Overall, OSA should be considered not only a predisposing factor but also a dynamic modulator of arrhythmic activity, capable of influencing both the initiation and persistence of rhythm disorders. Within a broader systems-level interpretation, intermittent hypoxia, oxidative stress, and inflammation may be viewed as interconnected regulatory processes that help sustain chronic electrical and structural instability over time, rather than as isolated downstream consequences of respiratory events alone [16]. This has important implications for patient management, as failure to recognize and treat sleep-disordered breathing may limit the effectiveness of conventional antiarrhythmic strategies.

## 4. Metabolic Modulation and Tirzepatide

The management of OSA-related arrhythmias increasingly extends beyond conventional approaches, with sustained weight reduction representing a key upstream strategy.

In this context, incretin-based therapies have emerged as important tools for achieving clinically meaningful weight loss, although their direct effects on arrhythmic outcomes remain insufficiently established. Among these agents, the dual glucose-dependent insulinotropic polypeptide (GIP) and glucagon-like peptide-1 (GLP-1) receptor agonist tirzepatide has demonstrated substantial and sustained reductions in body weight across multiple randomized trials, exceeding those observed with traditional GLP-1 receptor agonists [17]. However, it should be interpreted within the broader landscape of incretin-based and cardiometabolic therapies.

These effects extend beyond weight reduction, influencing multiple pathophysiological pathways relevant to OSA, including upper-airway mechanics, metabolic profile, and autonomic balance. Clinical studies have shown significant improvements in apnea–hypopnea index and nocturnal oxygenation parameters following pharmacologically induced weight loss, indicating a reduction in upper-airway collapsibility and hypoxic burden [18,19,20].

These changes may translate into downstream benefits on arrhythmic risk, although direct outcome-based evidence is still limited. By reducing visceral adiposity and possibly epicardial fat volume, metabolic therapies may attenuate local inflammation and myocardial fibrosis, while improvements in insulin sensitivity may contribute to a more stable electrophysiological environment. In addition, weight loss is associated with favorable modulation of autonomic tone, with reductions in sympathetic activity and restoration of physiological variability. Although direct evidence linking tirzepatide to arrhythmia reduction remains limited, the cumulative impact of these mechanisms provides a biological rationale for a potential role in modifying the arrhythmogenic substrate. This is consistent with previous observations showing that structured weight loss interventions reduce atrial fibrillation burden and improve outcomes following rhythm control strategies [21].

Metabolic therapy should be considered a potentially important component of the therapeutic framework in patients with obesity and OSA, particularly within a broader strategy of risk factor modification (Table 2).

From a practical perspective, the integration of metabolic therapy into routine care remains challenging and requires coordination between cardiology, sleep medicine, and metabolic specialists. However, direct evidence linking tirzepatide to arrhythmia reduction remains limited, and dedicated prospective studies are needed.

Therefore, while the mechanistic rationale is compelling, dedicated prospective studies are required to establish a direct causal relationship between tirzepatide therapy and arrhythmia reduction.

Tirzepatide should also be considered within the broader cardiometabolic therapeutic landscape. Other glucagon-like peptide-1 receptor agonists, such as semaglutide and liraglutide, have demonstrated clinically meaningful effects on body weight, glycemic control, and cardiovascular risk profile and may indirectly influence arrhythmic susceptibility through improvement in inflammation, insulin resistance, and autonomic function. In parallel, sodium–glucose cotransporter-2 inhibitors have emerged as relevant cardiometabolic agents, particularly in patients with heart failure and metabolic disease, although their direct effects on arrhythmia burden remain insufficiently defined. Overall, the available evidence supporting the antiarrhythmic benefits of these pharmacological strategies remains largely indirect, and dedicated studies with arrhythmic outcomes are still needed.

## 5. Cardioneuroablation

Cardioneuroablation (CNA) has emerged as an investigational therapeutic option for selected patients with functional bradyarrhythmias, particularly those mediated by excessive vagal activity. By targeting atrial ganglionated plexi, CNA aims to modulate cardiac autonomic input and reduce inappropriate parasympathetic influence on sinus and atrioventricular node function.

In patients with OSA, most bradyarrhythmias are transient and resolve with appropriate treatment of sleep-disordered breathing; however, a subset of patients may continue to experience persistent vagally mediated conduction disturbances despite optimal therapy.

In these cases, CNA may offer an alternative to permanent pacemaker implantation, particularly in younger individuals without structural conduction system disease. Several observational studies have reported favorable outcomes, including a reduction in syncope and bradyarrhythmic episodes, along with preservation of intrinsic cardiac rhythm [22,23]; however, the available evidence is still based predominantly on small cohorts and non-randomized data.

Recent advances in mapping and ablation technologies have refined the CNA workflow, improving both precision and reproducibility.

**Figure 4:**
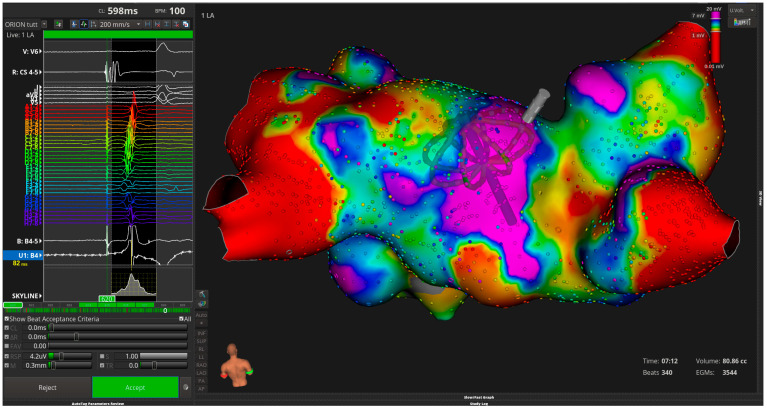
High-density electroanatomical map of the left atrium illustrating heterogeneous voltage distribution and fractionated electrograms. These findings reflect a complex atrial substrate and guide targeted ablation of autonomic inputs during cardioneuroablation procedures.

Ultra-high-density electroanatomical mapping allows detailed characterization of the atrial substrate. Automated electrogram analysis, sometimes supported by artificial intelligence, may facilitate the identification of fractionated signals corresponding to autonomic inputs (Figure 4).

In parallel, robotic magnetic navigation provides stable and atraumatic catheter control, enabling access to anatomically challenging regions with minimal fluoroscopy and more consistent lesion delivery. Together, these technologies may support a more targeted ablation strategy and may reduce procedural variability, although their availability remains limited to specialized centers, and their impact on long-term outcomes requires further validation [24].

From a procedural perspective, there is a growing shift toward biatrial approaches aimed at achieving more complete autonomic denervation. While right atrial ablation may be sufficient in selected cases, combined left and right atrial strategies appear to provide more durable modulation of both sinus and atrioventricular nodal inputs, at the expense of increased procedural complexity.

Patient selection remains essential. Ideal candidates typically exhibit clear evidence of vagally mediated bradyarrhythmias, preserved atrioventricular conduction outside symptomatic episodes, and no signs of intrinsic conduction system disease. The integration of clinical history, rhythm monitoring, and functional testing is therefore critical to identify those most likely to benefit from autonomic modulation.

Within the broader framework of the obesity–OSA–arrhythmia axis, CNA should be considered a targeted intervention for a specific clinical phenotype rather than a general therapeutic strategy. At present, its potential role appears most relevant in carefully selected patients with persistent, symptomatic vagally mediated bradyarrhythmias after optimization of respiratory and metabolic factors.

However, CNA should not be considered a substitute for etiological treatment of OSA but rather a complementary strategy in carefully selected patients with persistent, functionally mediated bradyarrhythmias. Randomized controlled trials and longer-term comparative data are still lacking, and this should be considered when interpreting the current role of CNA in routine clinical practice.

## 6. A Phenotype-Driven Approach to the Obesity–OSA–Arrhythmia Axis

**Figure 5:**
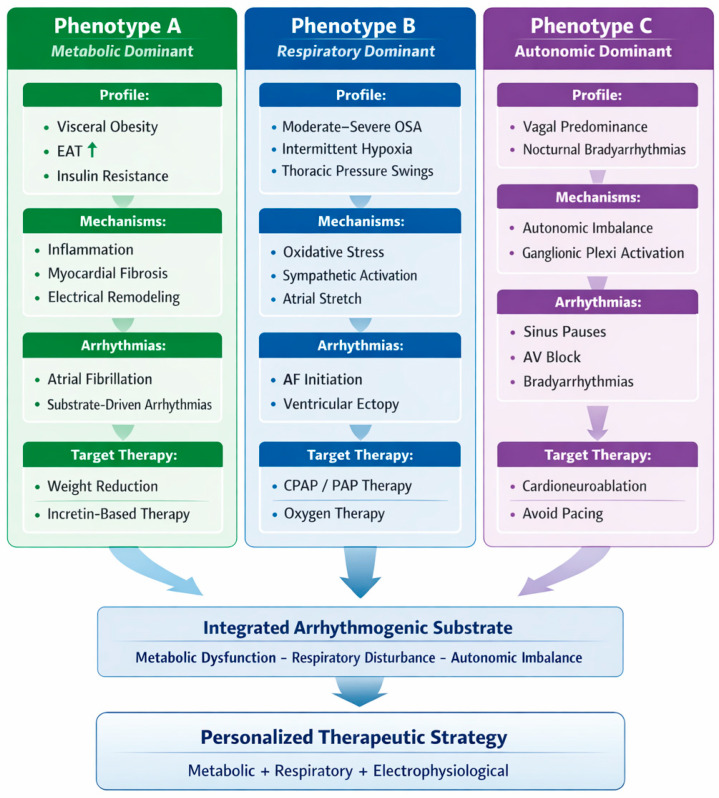
Patients can be stratified into three predominant phenotypes: metabolic, respiratory, and autonomic. Each phenotype is characterized by specific pathophysiological mechanisms contributing to arrhythmogenesis, including inflammation and fibrosis (metabolic), intermittent hypoxia and sympathetic activation (respiratory), and autonomic imbalance (autonomic). These mechanisms converge into a shared arrhythmogenic substrate, supporting a personalized therapeutic approach integrating metabolic, respiratory, and electrophysiological interventions.

The complex interplay between obesity, OSA, and cardiac arrhythmias is not uniform across patients but rather reflects a spectrum of pathophysiological profiles in which different mechanisms may predominate. In this context, a phenotype-driven approach may offer a more precise framework for understanding disease heterogeneity and guiding therapeutic strategies in individual patients (Figure 5).

Three main phenotypic patterns can be identified based on the predominant underlying mechanisms, namely metabolic-dominant, respiratory-dominant, and autonomic-dominant phenotypes, in line with emerging phenotype-based classifications in atrial fibrillation [25].

### 6.1. Metabolic-Dominant Phenotype

The metabolic-dominant phenotype is characterized by visceral obesity, increased epicardial adipose tissue, and insulin resistance. In this setting, arrhythmogenesis is primarily driven by chronic inflammation, adipokine imbalance, and myocardial fibrosis, which contribute to the structural and electrical remodeling of the atria. Patients with this phenotype are more likely to develop persistent forms of atrial fibrillation sustained by a well-established arrhythmogenic substrate. From a therapeutic perspective, interventions targeting weight reduction and metabolic modulation, including incretin-based therapies, represent key upstream strategies aimed at modifying the underlying substrate.

### 6.2. Respiratory-Dominant Phenotype

The respiratory-dominant phenotype is defined by moderate-to-severe OSA, with recurrent episodes of intermittent hypoxia and intrathoracic pressure fluctuations. In these patients, arrhythmias are often triggered by acute physiological stressors, including sympathetic activation, oxidative stress, and atrial stretch [26,27]. This phenotype is typically associated with the initiation of atrial fibrillation and the occurrence of nocturnal ventricular ectopy. Continuous positive airway pressure therapy and optimization of sleep-disordered breathing represent the cornerstone of management, with potential downstream effects on arrhythmic burden.

### 6.3. Autonomic-Dominant Phenotype

The autonomic-dominant phenotype is characterized by a predominance of vagal tone and functional bradyarrhythmias, often occurring during sleep and closely linked to apneic events. The underlying mechanism involves an imbalance in autonomic regulation, with enhanced parasympathetic activity mediated by ganglionated plexi. Clinically, this phenotype manifests as sinus pauses, sinus bradycardia, or atrioventricular conduction disturbances. In selected patients with persistent and symptomatic episodes despite optimal treatment of OSA, CNA may represent a targeted therapeutic option, allowing for modulation of autonomic inputs while avoiding unnecessary pacemaker implantation.

Importantly, these phenotypes are not mutually exclusive and may overlap within the same patient, contributing to the development of a complex and dynamic arrhythmogenic substrate. The relative contribution of each component may vary over time, influenced by disease progression and therapeutic interventions. This reinforces the need for an integrated and personalized approach combining metabolic, respiratory, and electrophysiological strategies, rather than relying on isolated therapeutic modalities.

From a practical clinical perspective, the proposed phenotype-driven framework should be interpreted as complementary to, rather than a substitute for, current guideline-based management. Contemporary atrial fibrillation guidelines already emphasize the importance of risk factor modification, including weight reduction and recognition of sleep-disordered breathing. In this context, phenotype-based stratification may help clinicians prioritize interventions according to the predominant pathophysiological driver in each patient, thereby improving the integration of metabolic, respiratory, and electrophysiological strategies in routine care.

## 7. Conclusions

The interplay between obesity, OSA, and cardiac arrhythmias reflects a complex and dynamic pathophysiological network rather than isolated disease entities.

Effective management requires a shift from symptom-oriented treatment to an integrated strategy targeting upstream drivers, including metabolic dysfunction, visceral adiposity, and sleep-disordered breathing.

Within this framework, interventions such as weight reduction, metabolic therapies, and selected electrophysiological approaches should be tailored to individual patient phenotypes. Future research should aim to better define patient selection and optimize the integration of these strategies into clinical pathways.

This phenotype-oriented model supports a shift toward precision medicine, in line with emerging phenotype-guided approaches in atrial fibrillation management.

Despite growing insight into the obesity–OSA–arrhythmia axis, several aspects remain incompletely defined, including the causal contribution of EAT to arrhythmogenesis, the extent to which metabolic therapies modify arrhythmic outcomes, and the identification of patients most likely to benefit from autonomic interventions such as CNA. A more systematic evaluation of negative or neutral findings from available studies would further help to refine the clinical applicability of this framework. Future prospective studies are needed to validate phenotype-driven therapeutic strategies and determine whether this integrated approach translates into improved long-term cardiovascular outcomes.

## Figures and Tables

**Table 1:** OSA-related arrhythmias: mechanisms, clinical features, and management.

Arrhythmia Type	Mechanisms in OSA	Clinical Features	Management Implications
**Atrial fibrillation**	Intermittent hypoxia, atrial stretch, inflammation, epicardial adipose tissue, autonomic imbalance	Increased incidence, persistence, and recurrence after ablation	PAP therapy, weight reduction, metabolic therapy, catheter ablation
**Ventricular arrhythmias**	Hypoxia-induced electrical instability, sympathetic activation, oxidative stress	Nocturnal clustering, association with desaturation events	Risk stratification, treatment of OSA, management of underlying heart disease
**Bradyarrhythmias**	Exaggerated vagal activation during apneic events	Sinus pauses, sinus bradycardia, atrioventricular block during sleep	PAP therapy, avoid unnecessary pacing, consider cardioneuroablation in selected cases

**Table 2:** Therapeutic strategies targeting the obesity–OSA–arrhythmia axis.

Target Level	Intervention	Mechanism of Action	Clinical Impact
**Respiratory (OSA)**	Positive airway pressure (PAP)	Reduces intermittent hypoxia and intrathoracic pressure swings	↓ arrhythmic burden, improved autonomic balance
**Metabolic (obesity)**	Lifestyle modification	Weight loss, reduced visceral and epicardial adiposity	↓ inflammation, improved metabolic profile
**Metabolic (pharmacological)**	Tirzepatide and incretin-based therapies	Weight reduction, improved insulin sensitivity, reduced OSA severity	Potential reduction in arrhythmic risk

## Data Availability

No new data were created or analyzed in this study.

## Abbreviations

The following abbreviations are used in this manuscript:

AF	Atrial fibrillation
AHI	Apnea–hypopnea index
AV	Atrioventricular
CNA	Cardioneuroablation
CPAP	Continuous positive airway pressure
CT	Computed tomography
EAT	Epicardial adipose tissue
GLP-1	Glucagon-like peptide-1
GIP	Glucose-dependent insulinotropic polypeptide
HFpEF	Heart failure with preserved ejection fraction
OSA	Obstructive sleep apnea
PA-TDI	Tissue Doppler-derived atrial conduction time
PAP	Positive airway pressure
